# Antibiotic susceptibility of orofacial infections in Bratislava: a 10-year retrospective study

**DOI:** 10.1007/s00784-024-05937-3

**Published:** 2024-09-21

**Authors:** Michal Vavro, Bronislava Dvoranová, Ladislav Czakó, Kristián Šimko, Branislav Gális

**Affiliations:** Faculty of Medicine, Department of Oral and Maxillofacial Surgery, Comenius University Bratislava, University Hospital Bratislava - Ružinov, Ružinovská 6 Bratislava 826 06, Bratislava, Slovakia

**Keywords:** Orofacial infections, Odontogenic abscess, Antibiotic resistance

## Abstract

**Objectives:**

Aim of this study was to analyse causal microbiological agents and their bacterial resistance in orofacial infections requiring hospital admission.

**Materials and methods:**

Presented is a 10-year retrospective study of patients hospitalised at a single department in 2014–2023. 744 patients were involved. In the statistical analysis, following data was evaluated: causal microbes and their resistance to Penicillin, Amoxicillin-Clavulanate, Clindamycin and Metronidazole.

**Results:**

Most frequent aetiology was odontogenic with causal tooth in socket (*n* = 468; 62,9%), followed by odontogenic – post extraction (*n* = 152; 20.4%), jaw fracture (*n* = 41; 5.5%), sialadenitis *n* = 31 (4.2%), osteonecrosis *n* = 22 (3.0%), oncological diagnosis in head and neck (*n* = 17; 2.3%), unknown (*n* = 10; 1.3%) and multiple factors (*n* = 3; 0.4%). 408 patients (54.8%) underwent extraoral abscess revision, 336 patients (45.2%) patients were treated locally without extraoral revision. In odontogenic group with tooth still present, superior CRP (m = 145.8 mg/l; SD = 117.7) and leukocyte values (m = 13.6*10^9^l; SD = 6.6) were observed in comparison to other groups. There were 698 cultivated bacteria in 362 patients. Most frequent bacteria were Streptococci (*n* = 162; 23.2%), Prevotella (*n* = 83; 11.2%) and Parvimonas (*n* = 65; 9.3%). Clindamycin resistance was highest (*n* = 180 resistant bacteria; 25.8%), followed by Metronidazole (*n* = 178; 25.5%), Penicillin (*n* = 107; 15.3%) and Amoxicillin-Clavulanate (*n* = 34; 4.9%).

**Conclusions:**

Orofacial infections in head and neck region are mostly of odontogenic origin with causal tooth still in socket. Causal bacteria show a high antibiotic resistance rate, especially to Clindamycin and Metronidazole.

**Clinical Relevance:**

Acquired data will be used to determine guidelines for empirical antibiotic prescription in cases of orofacial infections.

## Introduction

Orofacial infections are one of the most common infections of the head and neck region. These polymicrobial infections originate from the upper aerodigestive tract and can also involve deep neck spaces. Most common aetiological factor is odontogenic, often with failure of primary treatment – the causal gangrenous tooth still present in the socket [1–3]. The causative pathogens are in most cases oral commensal bacteria, such as gram-positive (G+) cocci or anaerobic gram-negative (G–) bacilli [4, 5]. Other causes of orofacial infections include salivary gland inflammation, untreated facial skeleton fractures, jaw osteonecrosis and tumours.

Dental infections should be treated locally by removing or trepanation of the causal tooth. Only in case of severe infections or complications, the treatment should involve antibiotics, which are in the beginning chosen empirically.

In the age of increasing antibiotic resistance, it is important to possess the knowledge of locoregional antibiotic resistance of most common pathogens found in abscess formations of the maxillofacial region. This information needs to be forwarded to the first attending physician, in this case, the general dentist [6]. 

Even though the evidence for antibiotics acting to prevent infection from surgical wounds in the mouth is poor to non-existent [7]. It has been noted that only about 12% of dentists adequately and correctly prescribe antibiotics, which shows the importance of comprehensive guidelines [8]. There are existing guidelines for antibiotic prophylaxis prescription, e.g. AHA 2007. However, according to published data [8], there is a need of guidelines not only for handling a cardiac or joint replacement patient, but also for regular oral surgery or dentistry procedures, or eventual complication handling, to prevent both over- and underprescription [9]. Nowadays, an overall overprescription of antibiotics is present – especially when they are being prescribed for all types of oral surgery, hugely increasing the risk of antibiotic resistance increase. Recommended antibiotics according to the authors are either Ampicillin or Amoxicillin-Clavulanate [10, 11].

Aim of this study was to evaluate the antibiotic sensitivity of bacteria cultured from orofacial space infections and to help choosing the right empirical therapy in the beginning of an abscess formation to avoid increasing bacterial resistance.

## Materials and methods

This is a 10-year retrospective study of patients admitted to the Department of Oral and Maxillofacial Surgery, Comenius University and University Hospital Bratislava in 2014–2023 with a diagnosis of an orofacial infection.

Study has been approved by Ethical Committee of University Hospital Bratislava – Ružinov (EK 079/2024).

### Statistical analysis

The collected data were subjected to statistical analysis in R Studio software to identify trends, correlations, and significant findings within the dataset. Descriptive statistics were used to summarize the data, and appropriate inferential statistics were applied to assess relationships between variables. The differences in C- reactive protein (CRP) and leukocyte count were tested among different aetiologies and localizations of abscess, using the Kruskal-Wallis rank sum test, as the data did not meet the assumptions of normality. Pairwise comparison between groups were performed with Bonferroni correction for multiple testing. To evaluate the relation between number of cultivated pathogens and values of CRP/leucocytes, two regression models were considered: a linear model, suggesting that a unit change in the number of pathogens corresponds to the same change in CRP/leukocyte values across all pathogens counts, and non-linear (categorical) model, in which number of pathogens is a categorical value and thus changes per unit change can differ (i.e., change from 0 to 1 pathogen present may affect CRP/leukocyte values differently than the change from 1 to 2 pathogens). The models were compared based on Akaike Information Criterion (AIC), with lower AIC indicating better fit. Difference in CRP and leukocytes between patients with resistant pathogen present and not present were tested using Kruskal-Wallis test.

### Data collection

The data were systematically collected from patient medical hospital records. These included: aetiology (the underlying cause of the orofacial infection), location (topographically specific anatomical location and side with the signs of orofacial infection), pathogen identification (the exact bacteria identified through laboratory microbiological analyses), antibiotic resistance (the resistance profile of the isolated pathogens, determined via the standardized antimicrobial susceptibility testing methods). Collected data was statistically evaluated.

### Treatment protocol

All patients in this study were admitted to hospital and were immediately administrated intravenous antibiotics. The first choice of antibiotics was Amoxicillin-Clavulanate in the dosage 1.2 g every 8 h, combined with intravenous Metronidazole 500 mg every 8 h. Patients allergic to Penicillin were administrated intravenous Clindamycin 600 mg every 8 h combined with intravenous Metronidazole 500 mg every 8 h. As soon as the result of microbiological swab was available and bacterial resistance to administered antibiotics was present, antibiotic type was changed. The odontogenic abscesses were treated by eliminating the odontogenic aetiology (tooth extraction) supported by intraoral incision in local anaesthesia, and in more severe cases, after CT confirming abscess formation, extraoral incision in general anaesthesia was performed. Drainage of the affected areas was performed. Plastic tubes were utilised for deep space drainage. These were rinsed regularly with 15% Betadine solution. After local and general improvement, the plastic tubes were extracted and exchanged to rubber drains until no liquid excretion was present.

## Results

Total number of patients was *n* = 744, 303 women and 441 men. The mean age was 43,1 years (SD 17,4).

Most frequent location was submandibular (*n* = 508; 68.3%), followed by perimandibular (*n* = 121; 16.3%), submental (*n* = 56; 7.5%), perimaxillary (*n* = 51; 6.9%) and buccal (*n* = 38; 5.1%). 30 patients had an infection of multiple deep spaces (4%).

Most frequent aetiology was odontogenic (*n* = 620; 83.3%). In 468 cases, the causal tooth was still present in the socket (62.9%), in 152 cases, the infection was a complication after tooth extraction (20.4%). Other aetiology included fracture of maxilla or mandible (*n* = 41; 5.5%), sialadenitis *n* = 31 (4.2%), osteonecrosis *n* = 22 (3.0%), oncological diagnosis in the head and neck (*n* = 17; 2.3%), in 10 cases, no specific cause was found (*n* = 10; 1.3%) and in 3 cases there were multiple factors present (*n* = 3; 0.4%). 408 patients (54.8%) underwent extraoral abscess revision, in 336 patients (45.2%) eliminating the cause in local anaesthesia was sufficient. (Chart no. 1).

**Chart no. 1:**
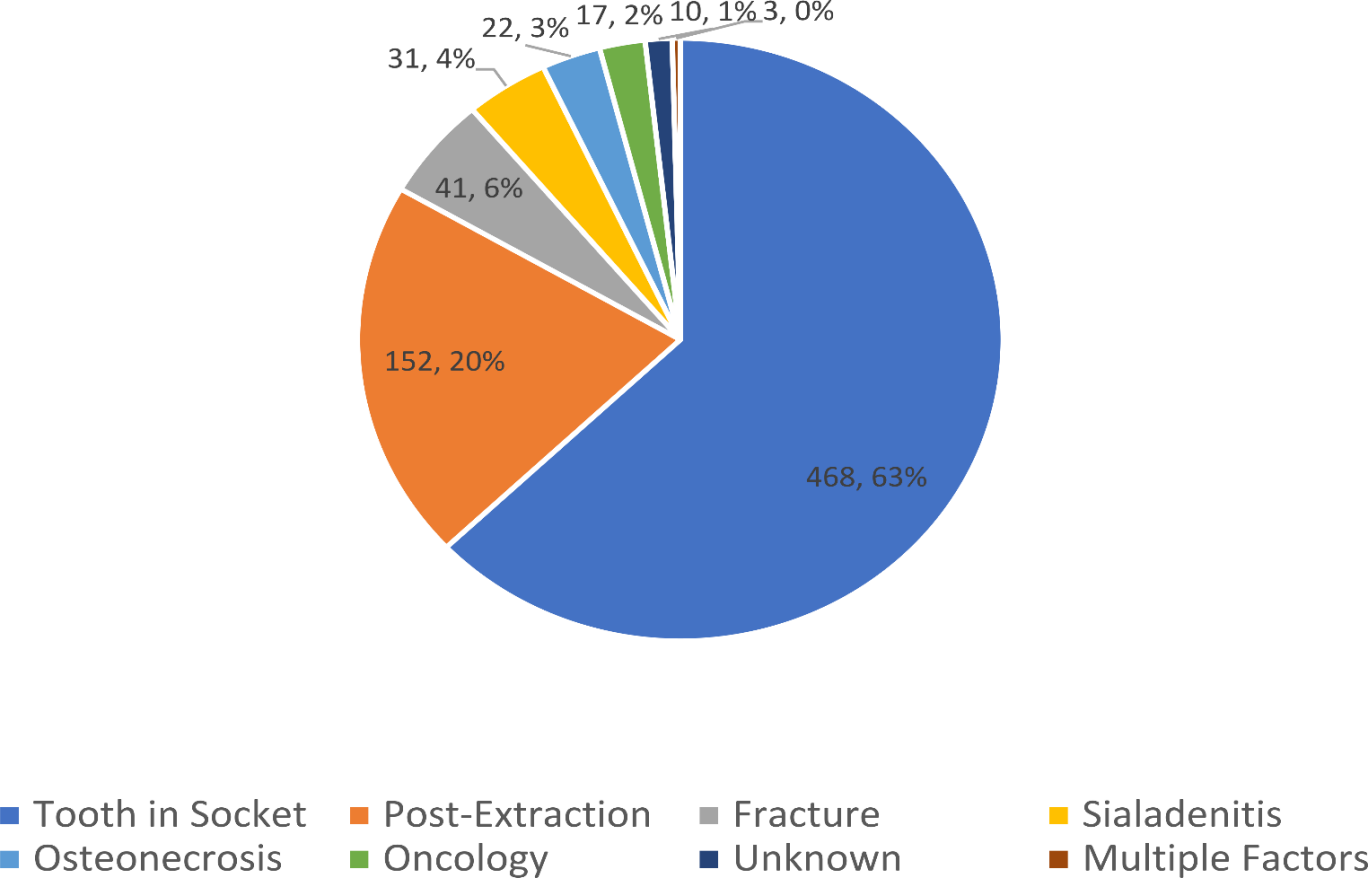
Orofacial Infection Aetiology

Statistically significant differences were observed in CRP values and leukocyte values between the aetiology groups (p < 0.001 for both CRP and leukocytes, Kruskal-Wallis rank sum test). Highest CRP (m = 145.8 mg/l; SD = 117.7) and leukocyte values (m = 13.6*10^9^l); SD = 6.6) were observed in group „*tooth in socket*.” (Chart no. 2,3). In pairwise comparison with Bonferroni correction for multiple testing, CRP in this group was significantly higher than in groups fracture (*p* < 0.001), oncology (*p* = 0.005), osteonecrosis (*p* = 0.017) and sialadenitis (*p* = 0.039). Similarly, leukocyte values in “tooth in socket” group were higher than those in groups fracture (*p* = 0.045), oncology (*p* = 0.019) and osteonecrosis (*p* < 0.001).

**Chart no. 2:**
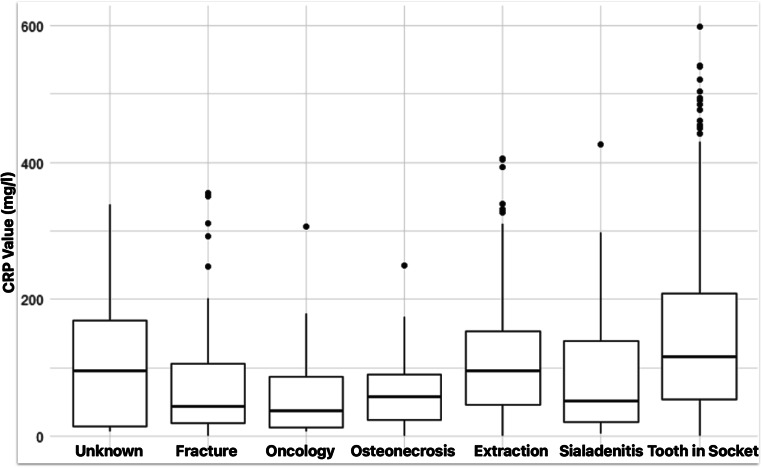
CRP Values in Groups Divided by Aetiology

**Chart no. 3:**
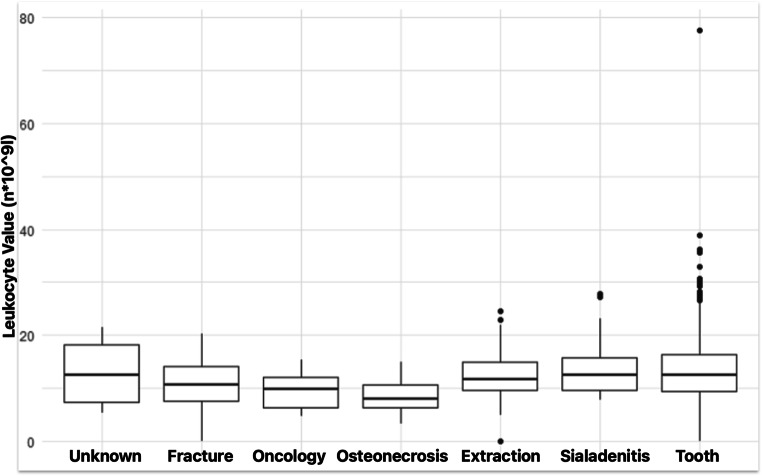
Leukocyte Values in Groups Divided by Aetiology

CRP values differed significantly among different localizations of deep space infections (*p* < 0.001, Kruskal Wallis rank sum test). Highest CRP values were observed in patients where multiple deep spaces were involved (m = 183.1 mg/l; SD = 144.9), followed by submandibular abscess group (m = 140.1 mg/l; SD = 112.9). (Chart no. 4)

**Chart no. 4:**
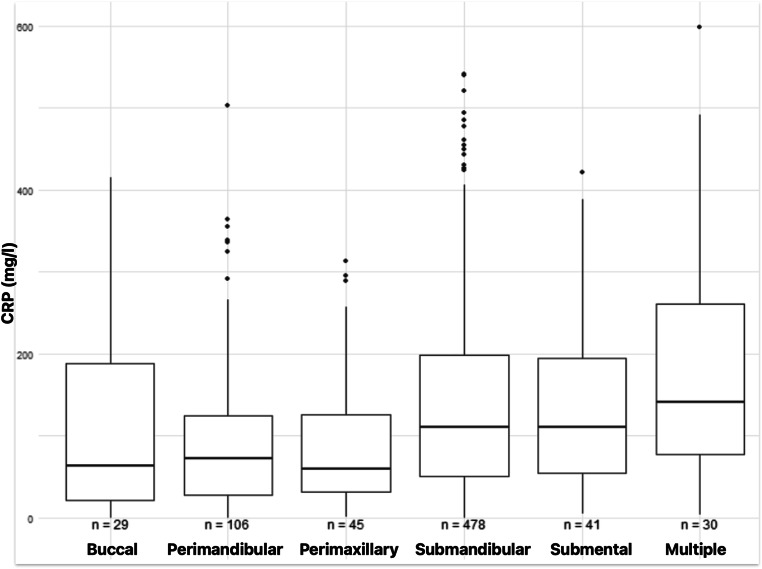
CRP Values in Groups Divided by Localization

Overall, there were 698 cultivated bacteria in 362 patients (in 382 patients, swab test turned out negative). The average bacteria count was 0.94 (SD 1.27), maximum bacteria count was 5. Most frequent bacteria were Streptococci (*n* = 162; 23.2%), Prevotella (*n* = 83; 11.2%) and Parvimonas (*n* = 65; 9.3%). (Table 1)

**Table 1:** All cultivated Bacteria

Bacteria	Count	Bacteria	Count
Abiotrophia	1	Haemophilus	11
Acinetobacter	2	Klebsiella	4
Actinobacillus	3	Lactobacillus	1
Actinotignum	1	Lancefieldella	2
Aggregatibacter	9	Micrococcus	4
Unidentified Alpha-Haemolytic	3	Morganella	1
Alloscardovia	1	Neisseria	1
Unidentified Anaerobic	13	Parascardovia	65
Arachnia	1	Parvimonas	1
Arthrobacter	1	Pasteurella	2
Atopobium	13	Peptococcus	2
Bacteroides	6	Peptoniphilus	9
Bifidobacterium	1	Porphyromonas	83
Campylobacter	4	Prevotella	19
Candida	6	Propionibacterium	1
Capnocytophaga	3	Proteus	3
Citrobacter	4	Pseudomonas	1
Corynebacterium	2	Rhizobium	2
Cutibacterium	39	Serratia	14
Delftia	1	Schalia	11
Dialister	10	Slackia	9
Eggerthia	5	Solobacterium	26
Eikenella	12	Staphylococcus	2
Enterobacter	10	Stenotrophomonas	162
Enterococcus	3	Streptococcus	10
Escherichia	1	Veillonella	1
Eubacterium	1	Ybiotrophia	1
Finegoldia	1	Acidaminococcus	3
Fusobacterium	19	Unidentified G+	1
Gemella	15	Unidentified G-	4
Granulicatella	14	**Total**	**698**

There was a statistically significant linear relation between CRP value of patient and number of cultivated pathogen (Adjusted R^2^ = 0.078, *p* < 0.001). The increase by 1 cultivated bacterium caused the increase of 24,75 mg/l in CRP of a patient. (Chart no. 5) Non-linear relationship was also considered and model with number of pathogens as a category was compared to linear model. However, in terms of AIC, the linear model was better at describing the relationship.

**Chart no. 5:**
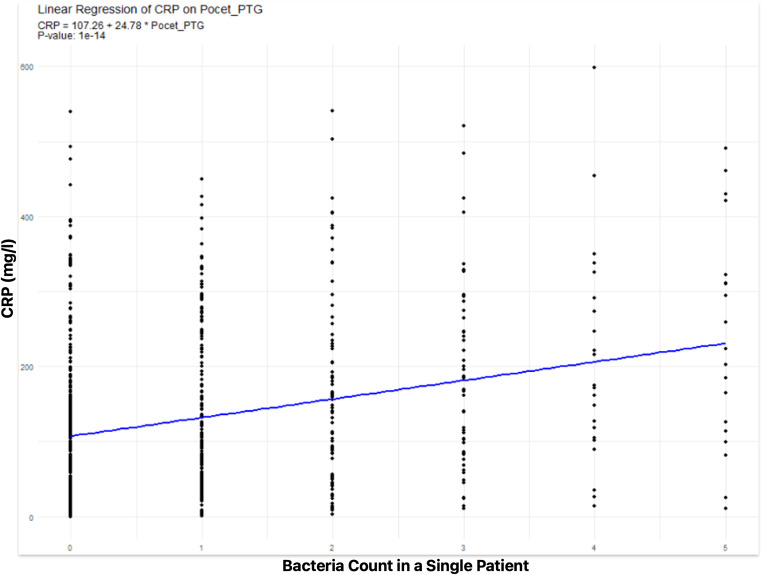
CRP Value Increase in Relation to Cultivated Bacteria Count

For closer evaluation, four antibiotics were chosen to evaluate their resistance rate: Penicillin, Amoxicillin-Clavulanate, Clindamycin and Metronidazole. Resistance was analysed as per cultivated bacteria. The most frequent was Clindamycin resistance (*n* = 180 resistant bacteria; 25.8%), next was Metronidazole (*n* = 178; 25.5%), Penicillin (*n* = 107; 15.3%) and Amoxicillin-Clavulanate (*n* = 34; 4.9%) (Chart no. 6).

**Chart no. 6:**
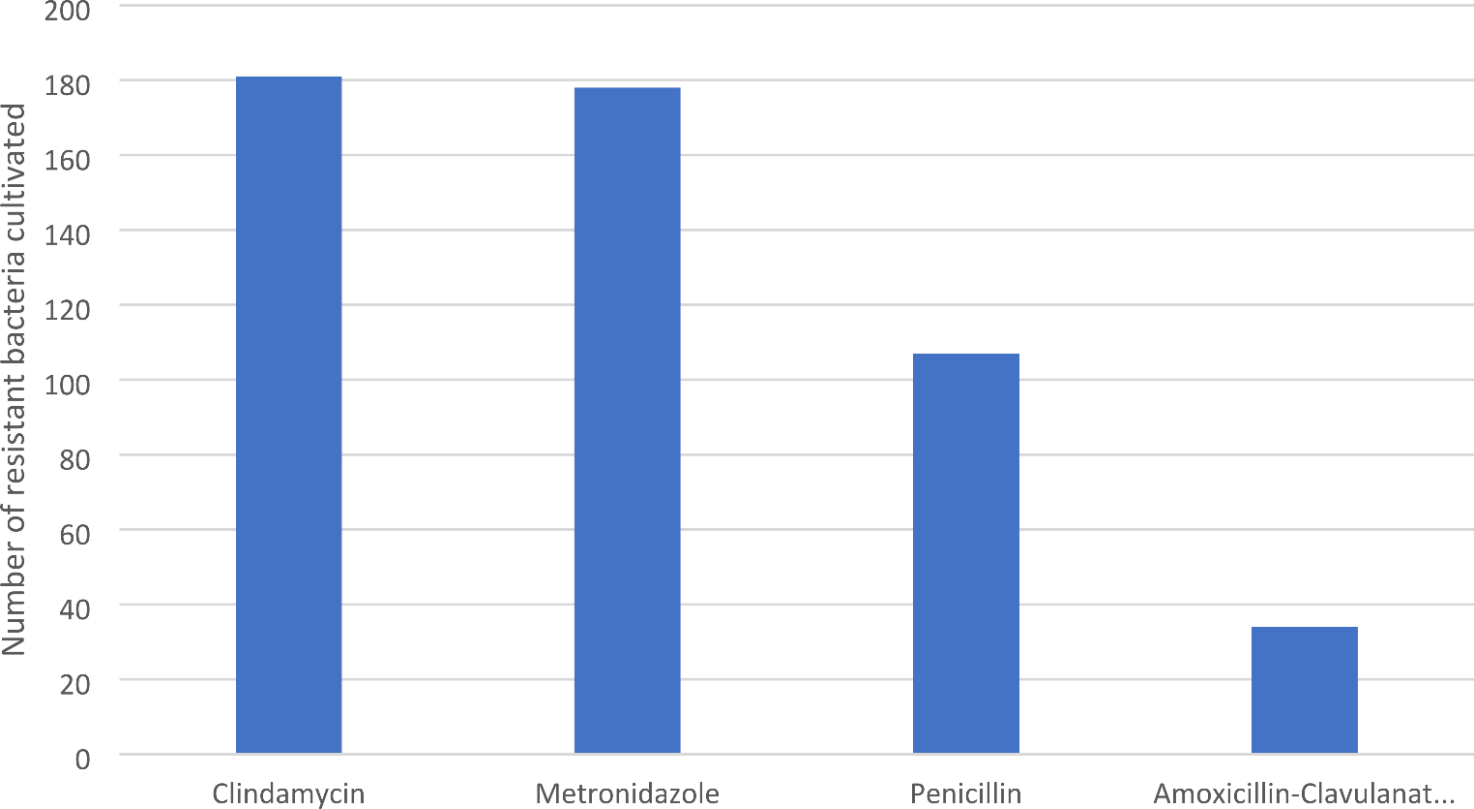
Antibiotic Resistance per Cultivated Bacteria

Patients presented with cultivated bacteria with resistance to at least one antibiotic type (*n* = 260) showed to have higher CRP values (158.4 mg/l; SD 123.8) compared to patients (*n* = 469) with bacteria showing no antibiotic resistance (114.4 mg/l; SD 101.7, *p* < 0.001, Kruskal-Wallis rank sum test).

When recounted to bacterial resistance per patient, 150 patients presented with a Metronidazole-resistant strain (41.4%), 141 patients with a Clindamycin-resistant strain (39%), 89 patients with a Penicillin-resistant strain (24.6%) and 28 patients with an Amoxicillin-Clavulanate resistant strain (7.7%) (Chart no. 7).

In the post extraction group, 45.6% patients with positive cultivation had bacteria resistant to Clindamycin present.

**Chart no. 7:**
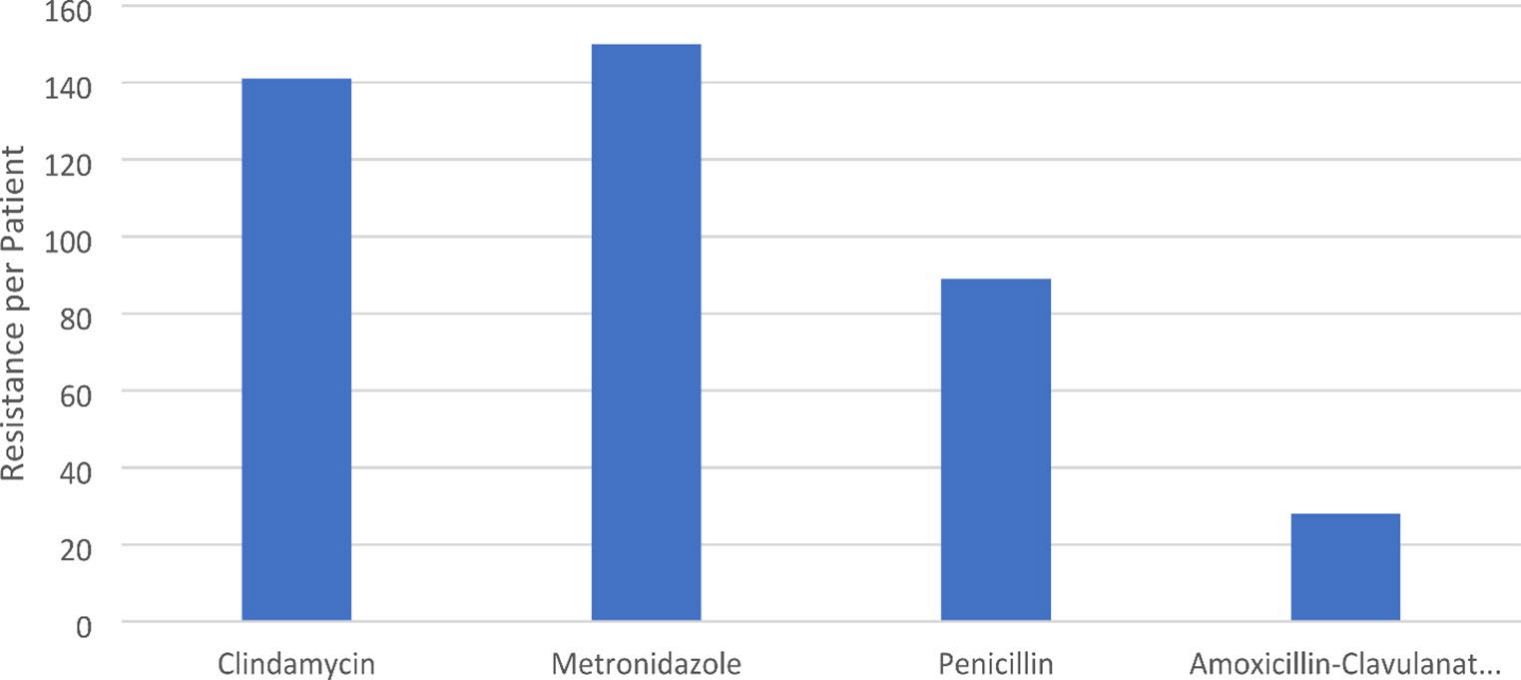
Bacterial Resistance per Patient

## Discussion

Antibiotic susceptibility and choice of empirical antibiotics in odontogenic abscesses is a subject of frequent discussion. There is a high frequency of antibiotic prescription among dentists, which can stand up to 10% antibiotic prescription in the whole country [12, 13]. Many authors agree there is a need of establishment of international guidelines [14].

The choice of antibiotics is empirical in the beginning of treatment. Therefore, it is important to be familiar with the most frequent causal bacteria and the most effective antibiotics to prescribe to capture the necessary spectrum.

Most authors agree Streptococci and other common oral flora such as Staphylococci and Prevotella as being the main bacterial strains, but the process is always a combined bacterial infection [4, 5]. For this kind of bacteria, Penicillin type antibiotics are recommended, preferably also with inhibitors of beta-lactamase [15–17], which is also confirmed by the results of this study. Cephalosporines can be used as an alternative to Penicillin [18, 19].

There has been a high resistance to Clindamycin reported in some studies [20, 21]. This high Clindamycin resistance was also present in this study (25.8%).

However, it is important to state that antibiotic treatment is secondary to surgical treatment. It is of utmost importance to remove the cause of the process and perform surgical evacuation and drainage of the process [15–17, 22, 23]. This has been confirmed by many studies comparing groups where antibiotics were administered in combination with surgical treatment, and where there was surgical treatment only [17, 22, 23]. When comparing these groups, the only difference in the outcome was a slightly longer hospital stay in the no antibiotics group [22].

Combined with the results of this study and considering the high bacterial resistance to administered antibiotics, it can be concluded the choice of antibiotics is not as important as the surgical procedure. When the real need for antibiotic therapy is detected, antibiotics should be used for the shortest time possible until the patient’s clinical cure is achieved [23].

## Conclusion

Orofacial infections in head and neck region are mostly of odontogenic origin, in most cases with the causal tooth still present in the socket. Odontogenic group with tooth still present in the socket also showed highest CRP and leukocyte values. Causal bacteria belong to physiological oral flora, consisting most frequently of Streptococci, Prevotella and Parvimonas. Cultivated bacteria show a high antibiotic resistance rate, especially to Clindamycin and Metronidazole. When prescribing antibiotics for orofacial infection empirically, it is recommended to choose Amoxicillin-Clavulanate.

## Data Availability

Data available on Synapse: 10.7303/syn61837109.1.
